# Saikokeishikankyoto extract alleviates muscle atrophy in KKAy mice

**DOI:** 10.1007/s11418-021-01590-2

**Published:** 2022-01-08

**Authors:** Yanglan Ou, Kohei Jobu, Tomoaki Ishida, Shumpei Morisawa, Hiroko Fujita, Kei Kawada, Saburo Yoshioka, Mitsuhiko Miyamura

**Affiliations:** 1 Graduate School of Integrated Arts and Sciences, Kochi University, 185-1 Kohasu, Oko-cho, Nankoku, Kochi Japan; 2 Department of Pharmacy, Kochi Medical School Hospital, 185-1 Kohasu, Oko-cho, Nankoku, Kochi Japan

**Keywords:** Muscle atrophy, Sarcopenia obesity, Saikokeishikankyoto, KKAy, Sirtuin1

## Abstract

Sarcopenic obesity is associated with increased visceral fat and decreased muscle mass, resulting in decreased insulin sensitivity, increased production of inflammatory cytokines, and oxidative stress. In this study, we first evaluated the effects of herbal medicines on the transcriptional activity of the Sirtuin 1 (*sirt1*) promoter in vitro as an indicator of their therapeutic effect. Our data suggested that hot water Saikokeishikankyoto (SKK) extracts increased *sirt1* transcriptional activity in vitro*,* identifying it as a candidate therapeutic for evaluation in the KKAy type 2 diabetic obesity mouse model. These in vivo evaluations revealed that SKK treatment increased the wet weight and muscle fiber content in cross sections of the gastrocnemius muscle (GA) and restored motor function in these animals. In addition, SKK treatment reduced tumor necrosis factor-α (TNFα) expression in the sera and suppressed *Atrogin1* and *MuRF1* transcription in the GA samples. This treatment also increased *sirt1* expression in these tissues. These results suggest that SKK inhibits skeletal muscle atrophy and improves motor function in KKAy mice by suppressing inflammation. In actual clinical practice, SKK is expected to inhibit muscle atrophy and improve motor dysfunction in sarcopenic obesity.

**Graphical abstract:**

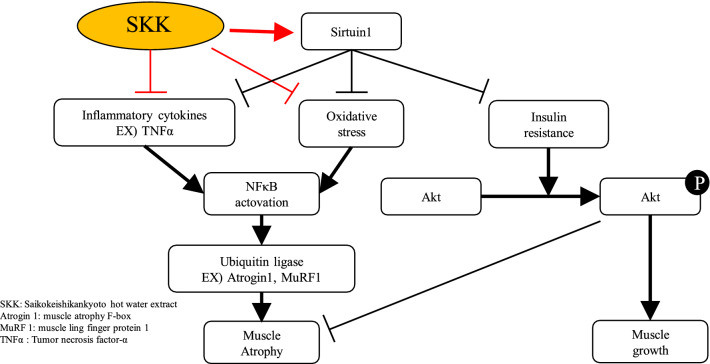

**Supplementary Information:**

The online version contains supplementary material available at 10.1007/s11418-021-01590-2.

## Introduction

Sarcopenic obesity is associated with increased visceral fat and decreased muscle mass [1, 2], and decreases the patient's motor function and significantly reduces quality of life [3, 4]. Currently, the main treatment for sarcopenic obesity is exercise therapy, not drug therapy. The symptoms of sarcopenic obesity have been reported to progress as a result of decreased insulin sensitivity and increased inflammation and oxidative stress [5–9]. Insulin promotes muscle protein production, but during sarcopenic obesity, sensitivity to insulin is reduced and muscle protein synthesis is decreased [5, 6]. In addition, inflammatory cytokines, such as TNF-α and IL-6, are produced in the adipose tissues [10, 11] increasing inflammation and resulting in the atrophy of the skeletal muscle via increased expression of the ubiquitin ligases [12, 13]. Oxidative stress also increases with the enlargement of the fat cells resulting from obesity and hyperglycemia [14]. Oxidative stress causes muscle cell apoptosis and degradation of the muscle fibers [15, 16] and these factors combine in sarcopenic obesity to produce significant skeletal muscle atrophy. KKAy is a robust murine model for type 2 diabetes that can be used to evaluate both early and severe obesity and hyperglycemia, resulting in decreased insulin sensitivity, overproduction of inflammatory cytokines, and increased oxidative stress, similar to the symptoms associated with sarcopenic obesity [17, 18].

Several studies have reported that various substances increase Sirtuin1 (*sirt1*) expression and exert an inhibitory effect on muscle atrophy [19, 20]. This is thought to be due to the fact that *Sirt1* suppresses the expression of ubiquitin ligase [21]. In addition, *Sirt1* has been reported to inhibit inflammation and oxidative stress [19], and improve insulin sensitivity [22]. Therefore, *sirt1* expression may confer a protective effect against the muscle atrophy associated with sarcopenic obesity. We focused on herbal medicines with components that have been shown to upregulate the expression of *sirt1*. These herbal medicines include several traditional Japanese medicines that consist of a combination of several herbs. Recently, several herbal medicines have been reported to inhibit muscle atrophy [23–26] suggesting that these therapeutic compounds may act as a preventive treatment for sarcopenia.

In this study, we evaluated the effects of 62 herbal medicines on the transcriptional activity of *sirt1* in C2C12 cells. We then evaluated the inhibitory effect of SKK, which was screened as the best-performing extract, on muscle atrophy using KKAy mouse model.

## Materials and methods

### Cell culture and differentiation of C2C12 cells

C2C12 cells were purchased from Riken (Tsukuba, Japan) and cultured in Dulbecco's modified Eagle's medium (DMEM; Wako, Osaka, Japan), supplemented with 10% fetal bovine serum (FBS; Biowest, Nuaille, France), 100 U/ml penicillin, and 0.1 mg/ml streptomycin (Nakarai, Kyoto, Japan). Cultures were maintained at 37 °C in a 5% CO_2_ incubator. At approximately 70% confluence, C2C12 cells were differentiated for 96 h in DMEM supplemented with 2% horse serum (Biowest, Nuaille, France). The medium was changed every 24 h until myotubes were observed.

### Preparation of herbal medicines for in vitro experiments

Hot water extracts of 62 herbal medicines [27] were provided to our group from Tsumura (Tokyo, Japan) as spray dried products. Each hot water extract (1.0 g) was dissolved in methanol (20 ml), ultrasonically extracted for 30 min, and centrifuged at 8000×*g* for 5 min, after which the supernatant was recovered before being concentrated under reduced pressure in an evaporator and dissolved at a concentration of 100 mg/ml in dimethyl sulfoxide (DMSO). These extracts were then stored at − 20 °C prior to use. Experiments consisted of dissolving an appropriate amount of each extract in the cell culture media. The concentration of DMSO in the medium was adjusted to less than 0.1%.

### Luciferase activity assay

C2C12 cells were seeded in 9.5-cm dishes and then transfected with the pCMV-LacZ (Takara Bio, Otsu, Japan) and pTA Luc-Sirt 1-promoter (Add gene, Cambridge, USA) [28] plasmids using Hily MAX (Tokyo Doujin, Tokyo, Japan). Transfected cells were then treated with a 0.25% trypsin solution and seeded in 24-well plates, at 2.0 × 10^4^ cells/well, and incubated for 72 h in differentiation medium. The medium was then replaced with the media containing the herbal medicine extracts or resveratrol (20 μM) and then evaluated. Resveratrol (Wako, Tokyo, Japan) is a *sirt1* activator and was used as a positive control for all of our in vitro experiments. After 12 h of culture the medium was removed, and the cells were washed with PBS and then lysed. We then measured the luciferin fluorescence in each well and the fluorescence intensity was corrected using the pCMV-LacZ plasmid as an internal standard. The data were reported as the ratio of the sample to the chemiluminescence of the control group. The amount of extract contained in the daily dose was defined as 1 unit (U) and all 62 herbal medicine extracts were added to the C2C12 cells at a concentration of 1.0 × 10^–4^ U/ml. In a similar manner, *sirt1* promoter activities of SKK at each concentration (30, 100, 1000 μg/mL) and crude drugs consist of SKK were measured. For the preparation of the constituent crude drug samples of SKK, each constituent crude drug of the daily dose of SKK was extracted with hot water, and the methanol-solubilized portion was extracted. Each constituent crude drug was used in the *sirt1* promoter assay at a dose of 1.0 mg/ml, corresponding to the methanol-solubilized portion of SKK (Table 1).

**Table 1:** Crude drugs of SKK and extraction efficiency

Name of formulation and crude drug	Japanese name	Ratio (g/day)	Hydrothermal extraction efficiency (%)	Extraction efficiency of methanol soluble part (%)	Equivalent to 1.0 mg/ml of SKK methanol soluble (μg/mL)
Saikokeishikankyoto	Saikokeishikankyoto	22	46.7	41	–
Bupleuri radix (root of *Bupleurum falcatum*)	Saiko	3	25.9	53.2	291
Scutellariae radix (root of *Scutellariae baicalensis*)	Ougon	3	42.8	12.6	114
Cinnamomi cortex (bark of *Cinnamomum cassia*)	Keihi	3	15.7	41.4	137
Trichosanthis radix (root of *Trichosanthes kirilowii*)	Karokon	6	20.4	29.5	254
Ostreae testa (shell of Ostrea gigas)	Borei	3	0.5	37.7	4
Zingiberis rhizoma (root of *Zingiber officinale*)	Kankyo	2	20.2	30.5	87
Glycyrrhizae radix (root of *Glycyrrhiza uralensis*)	Kanzo	2	19.8	40.5	113

### Preparation of SKK feed

The SKK [27] was provided as a spray-dried product from Tsumura (Tokyo, Japan). A total of 5 g of hot water extract was obtained from the 22 g crude drug mixture, as shown in Table 1. SKK was mixed with normal feed (CE-2, CLEA, Shizuoka, Japan) at a concentration of 2% or 4% w/w and the 3D-HPLC spectrum for SKK is shown in the Supporting Information. In addition, the murine diets were mixed with 0.4% starch as a negative control and 0.4% resveratrol as a positive control. The resveratrol dosage was determined using the quantities applied in previous studies [29, 30].

### Animals

Animal experiments were conducted with permission from the Kochi University Animal Experiment Ethics Committee (Approval No. M-00031, approval date: June 21, 2019) and the KKAy mice were purchased from Japan CLEA (Kanagawa, Japan).

The 8-week-old KKAy mice (male) were randomly divided into four groups, and each group received either control (CE-2 feed), 2% SKK-mixed feed, 4% SKK-mixed feed, or 0.4% resveratrol feed. Each mouse was then observed over the next 42 days.

### Rotarod performance test

In the rotarod test, the speed of the rotor was increased at a rate of 0.3 rotations/second until it reached a maximum of 80 rotations/min. We then measured the time until the mouse fell for each group. Each mouse was tested three times at 10-min intervals, and the mean of these three tests was used as the result. The rotarod test was conducted every 7 days over the 42-day experimental period.

### Food intake, body weight, blood glucose, skeletal muscle weight, and TNF-α concentration measurements

Dietary intake was measured every 3–4 days at the time of dietary change and the data was consolidated every 7 days. The mice underwent laparotomy under anesthesia 42 days after starting SKK treatment and their body weight and blood glucose concentration were measured. The mice were fasted for 6 h prior to the blood glucose measurement and the blood was collected from the tail vein and tested using Glu-TEST-STRIPS (Nova Biomedical, Tokyo, Japan). Whole blood samples were collected from the descending vena cava. The gastrocnemius (GA) and the soleus muscles were extracted from the lower limbs before the wet weight of each muscle tissue was measured. The serum concentration of TNF-α was determined using a TNF-α ELISA kit (R&D Systems, Minneapolis, MN, USA).

### Measurement of myocyte cross-sectional area

The excised gastrocnemius muscle was fixed with 10% formalin, embedded in paraffin, sectioned at 5 μm, and stained with hematoxylin and eosin. A cross section of the gastrocnemius muscle was traced using the computer analysis software WinROOF (Mitani Corporation, Ohtsu, Japan), and the cross-sectional area of each fiber was measured. The cross-sectional areas of 200 muscle fibers from each mouse were randomly measured, and the average value was used in our downstream analysis.

### Quantitative reverse transcription polymerase chain reaction (qRT-PCR)

Total RNA was collected from GA using the RNeasy Fibrous Tissue Mini Kit (Qiagen, Hilden, German). Thereafter, reverse transcription was carried out using the PrimeScript RT reagent Kit (Takara Bio, Otsu, Japan). qRT-PCR of the mRNA in the GA samples was performed as previously described [25]. qRT-PCR was performed using the Step One Plus Real-Time PCR System (Applied Biosystems, CA, USA) and the mRNA expression levels of *sirtuin1* (*sirt1*) and ubiquitin ligases (*murf1* and *atrogin1*) were evaluated using *GAPDH* as the internal reference standard. TaqMan probes: *GAPDH* (Mm99999915_g1), *sirt1* (Mm01168521_m1), *muscle RING-finger protein-1* (*murf1*) (Mm01185221_m1), and *atrogin1* (Mm00499523_m1) were used in combination with TaqMan Universal PCR Master Mix (Applied Biosystems, Foster City, CA, USA).

### Statistical analysis

The results are expressed as the mean ± standard deviation (S.D). We evaluated the rotarod test results using a two-way repeated analysis of variance (ANOVA), while all of the other data were evaluated using one-way ANOVA. We then evaluated any statistically significant (*P* < 0.05) *F* values using Tukey’s test. Statistical analysis was performed using the Stat Flex program (View Flex, Tokyo, Japan) and statistical significance was set at *P* < 0.05.

## Results

### Changes in *sirt1* promoter following treatment with 62 herbal medicines

**Fig. 1:**
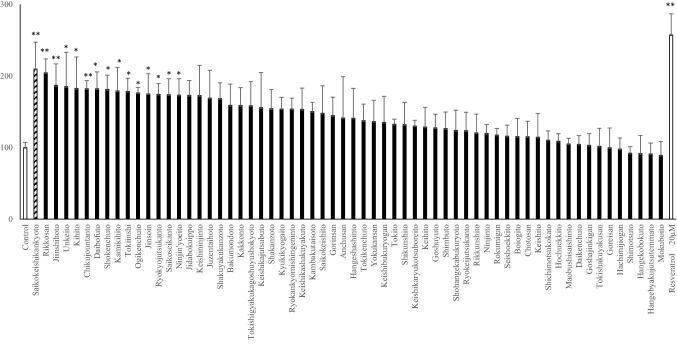
Herbal medicine extracts on *sirt1* promoter activity in C2C12 cells. C2C12 cells were treated by each herbal medicine extracts for 12 h, and *sirt1* promoter activity in each cells were measured. The amount of extract contained in the daily dose was defined as 1 U and the extract was added to C2C12 cells at a concentration of 1.0 × 10^–4^ U/ml. These data were expressed as mean ± SD (*n* = 4), and evaluated by one way ANOVA, and followed by Bonferroni test. *P < 0.05, **P < 0.01 vs control group

We evaluated the effects of each of the 62 herbal medicine extracts in C2C12 cells using a luciferase assay (Fig. 1). This assay allowed us to evaluate the transcriptional activity of the *sirt1* promoter under these conditions and showed that both the SKK and Rikkosan hot water extracts induced an almost 200% increase in *sirt1* promoter activity compared to the control (no treatment). Closer evaluation revealed that the transcriptional activity of *sirt1* was highest in the SKK treated cells.

### *sirt1* promoter activity in C2C12 cells

**Fig. 2:**
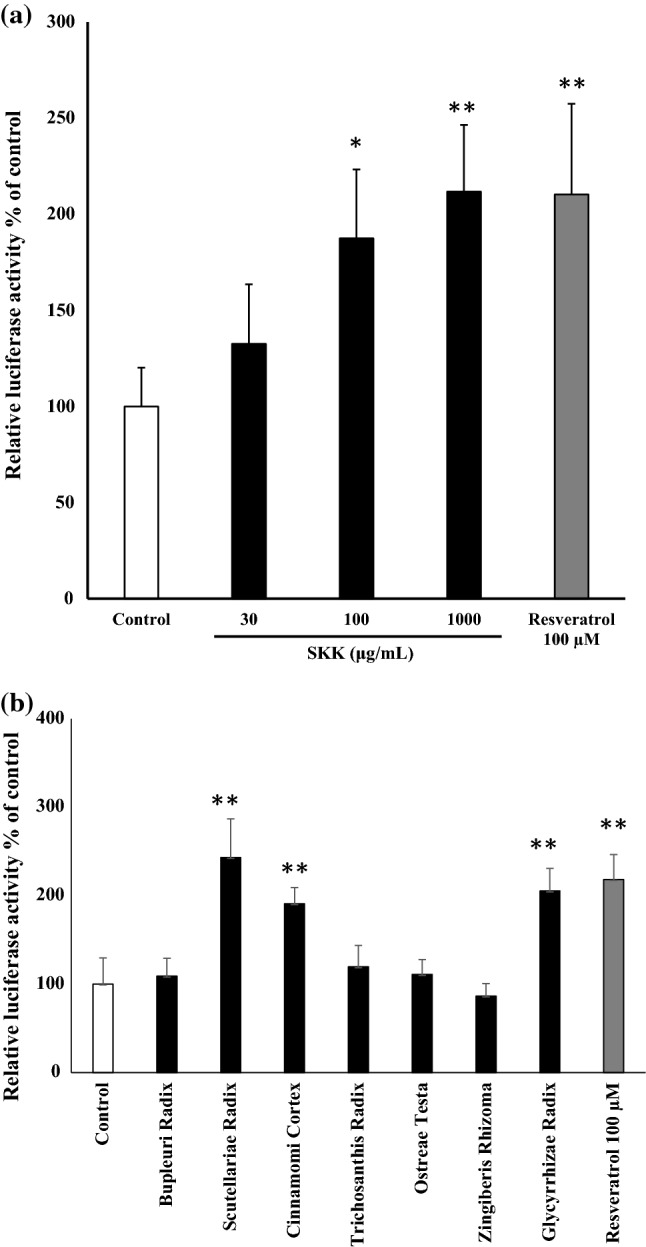
Effect of SKK and its component crude drugs on *sirt1* promoter activity in C2C12 cells. C2C12 cells were treated **(a) **SKK at each concentration (30, 100, 1000 μg/mL) or **(b)** crude drug equivalent to 1.0 mg/mL in soluble portion of SKK methanol for 12 h, and *sirt1* promoter activity in each cells were measured. Resveratrol (100 μM) was used as positive control. These data were expressed as mean ± SD (*n* = 4), and evaluated by one-way ANOVA, and followed by Tukey’s test. *P < 0.05, **P < 0.01 vs. control group

We evaluated the effects of SKK and crude drugs consist of SKK in C2C12 cells using a luciferase assay. *sirt1* promoter activities were significantly higher in the 100 μg/ml SKK (*P* < 0.05), 1000 μg/mL SKK (*P* < 0.01), and resveratrol groups (*P* < 0.01) when compared to the control (Fig. 2a).

The results also suggested that *sirt1* promoter activity and SKK concentration tend to be correlated. In the Crude drugs of SKK, *Scutellariae Radix*, *Cinnamomi Cortex*, and *Glycyrrhizae Radix* groups were significantly higher in *sirt1* promoter activity than the control (*P* < 0.01) (Fig. 2b).

### Food intake, body weight, blood glucose levels, and skeletal muscle weight

There was no significant difference in dietary intake between all groups for all time periods. (Supplementary information 3) Body weight, blood glucose levels, and skeletal muscle weight were measured after 42 days of SKK treatment (Table 2). There was no significant difference in body weight among the groups but blood glucose levels were significantly lower in the 4% SKK (*P* < 0.05) and resveratrol groups (*P* < 0.01) when compared to the control. In addition, the extensor digitorum longus was significantly heavier in the 4% SKK (*P* < 0.05) and resveratrol groups (*P* < 0.01) when compared to the control, and the gastrocnemius muscle was significantly heavier in the 4% SKK (*P* < 0.05), 2% SKK (*P* < 0.05), and resveratrol groups (*P* < 0.01) compared to the control. There were no differences in the soleus muscles in any of these groups.

**Table 2:** Effect of SKK for body weight, blood glucose concentration, and skeletal muscle weight

	Control	2% SKK	4% SKK	Resveratrol
Body weight (g)	44.7 ± 1.1	43.3 ± 2.0	42.5 ± 0.9	43.2 ± 1.2
Blood glucose (mg/dL)	424 ± 75	384 ± 65	299 ± 28*	287 ± 81*
Gastrocnemius (mg)	129 ± 4	134 ± 7	142 ± 7*	149 ± 9**
Extensor digitorum longus (mg)	14.7 ± 1.8	17.3 ± 1.9*	17.5 ± 1.1*	18.3 ± 1.4*
Soleus (mg)	6.7 ± 2.1	7.1 ± 1.7	7.6 ± 1.2	6.8 ± 0.7

Skeletal muscles were removed from mice at 42 days after the start of SKK administration. And, these wet weight were measured. These data were expressed as mean ± SD (*n* = 7), and evaluated by one way ANOVA, and followed by Bonferroni test. *P < 0.05, **P < 0.01 vs control group

One-way ANOVA revealed that these effects were significant (body weight: *F*_3,24_ = 9.8, *P* < 0.01; blood glucose: *F*_3,24_ = 8.0, *P* < 0.01; gastrocnemius: *F*_3,24_ = 11.3, *P* < 0.01; extensor digitorum longus: F_3,24_ = 7.8, *P* < 0.01; soleus: *F*_3,24_ = 0.32, *P* = 0.81).

### Rota-rod test

**Fig. 3:**
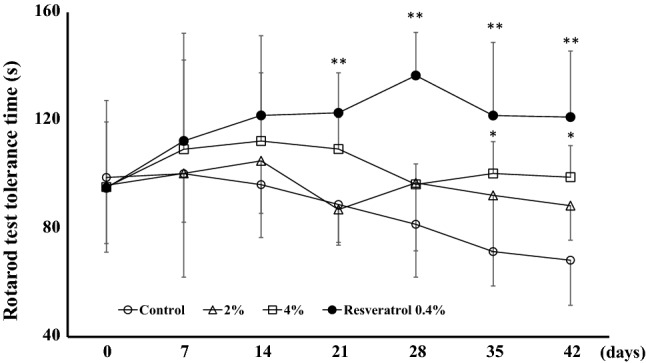
SKK treatment improves motor function. We used the rotarod tolerance test to evaluate changes in motor function following 42 days of treatment with SKK. These data are expressed as the mean ± SD (*n* = 7) and evaluated by two-way ANOVA, followed by Tukey’s test at for each timepoint. *P < 0.05, **P < 0.01 vs. the control group

Subsequently, motor function in these animals was evaluated using the Rota-rod test. These evaluations were completed every 7 days from the start of SKK administration, and the time until the mouse dropped from the rotor was measured. There was no difference in endurance time between the groups at day 0, but the endurance time of the rotor-rod test gradually decreased with time in the control animals. However, both the 4% SKK and resveratrol groups exhibited significantly improved endurance times when compared to that of the control group [4% SKK group vs. control group: day 35 (*P* = 0.044) and day 42 (*P* = 0.013), resveratrol group vs. control group: day 21 (*P* < 0.01), day 35 (*P* < 0.01), and day 42 (*P* < 0.01)] (Fig. 3).

Two-way ANOVA indicated that these effects were significant with respect to time (*F*_6,168_ = 2.3, *P* = 0.037), group (*F*_3,168_ = 6.5, *P* < 0.01), and interaction between time and group (*F*_18,168_ = 2.0, *P* = 0.013).

### Myocyte cross-sectional area

**Fig. 4:**
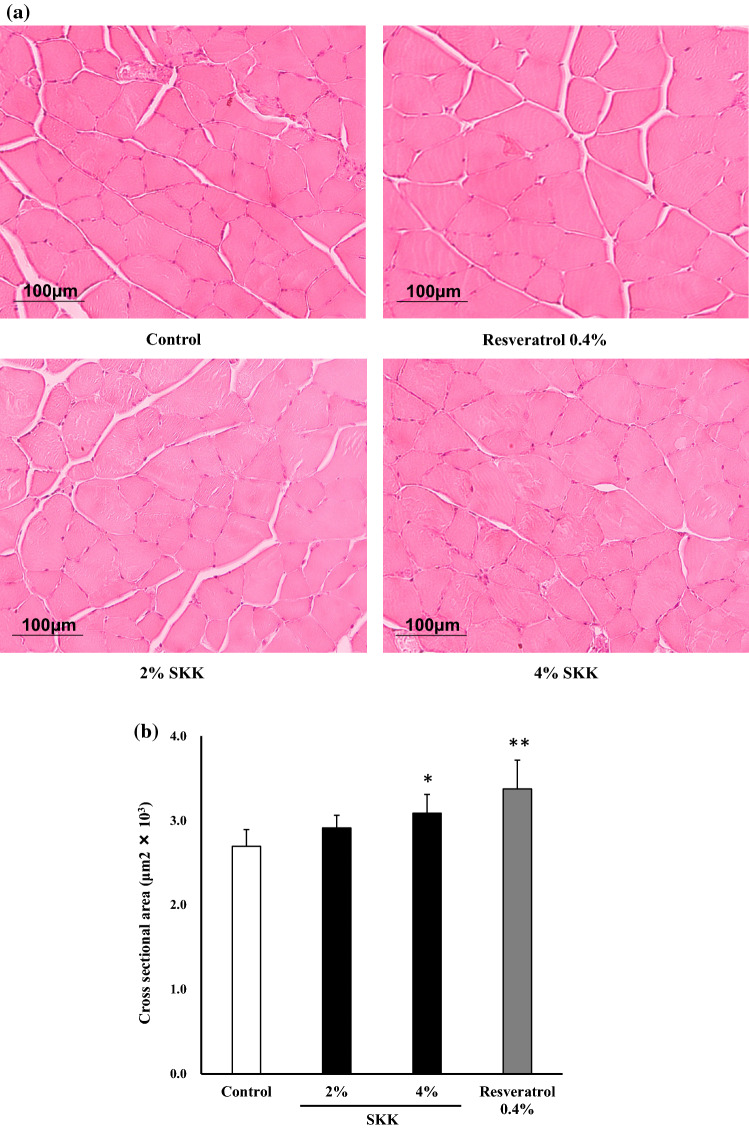
Skeletal muscle atrophy in mice. The gastrocnemius muscle was removed from each animal 42 days after the start of SKK administration and then stained with hematoxylin and eosin. **a** Myocyte cross sections of the gastrocnemius, **b** summary data for the myocyte cross sections. These data are expressed as the mean ± SD (*n* = 7) and evaluated by one-way ANOVA and Tukey’s test. *P < 0.05, vs. the control group

The cross-sectional area of the GA muscle fibers extracted after 42 days of SKK treatment were compared to those of the other groups in an effort to confirm the protective effect of this treatment for skeletal muscle tissues. The GA muscle fiber cross-sectional areas from both the 4% SKK (*P* = 0.025) and resveratrol groups (*P* < 0.01) were shown to be significantly increased in comparison to the control group (Fig. 4a, b) and one-way ANOVA indicated that this effect was significant (*F*_3,24_ = 10.1, *P* < 0.01).

### Circulating inflammatory cytokines

**Fig. 5:**
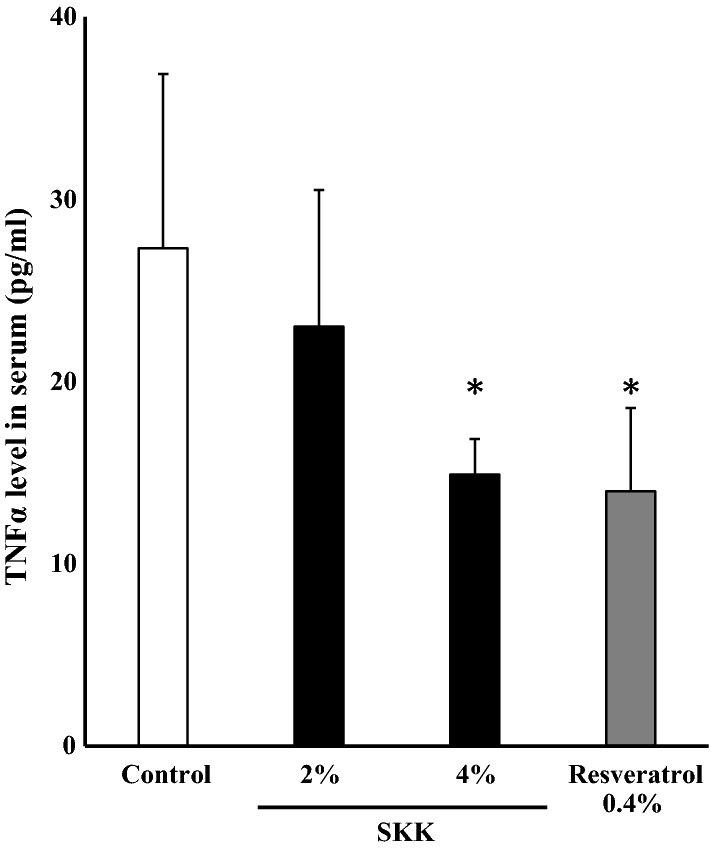
SKK treatment decreased TNFα concentrations in the serum. Blood was collected from each mouse 42 days after the start of SKK administration and then used to evaluate the TNFα concentration levels in the serum. These data are expressed as the mean ± S.D. (*n* = 7) and evaluated by one-way ANOVA and Tukey’s test. *P < 0.05, vs. the control group

The level of TNF-α expression in the serum samples from mice treated with SKK was evaluated to determine the inhibitory effect of SKK on inflammatory cytokines. Serum TNF-α levels in both the 4% SKK group (*P* < 0.01) and resveratrol groups (*P* < 0.01) were shown to significantly decrease compared to the control group (Fig. 5), with these values supported by our one-way ANOVA (*F*_3,24_ = 7.5, *P* < 0.01).

### *sirt1* gene expression in skeletal muscle

**Fig. 6:**
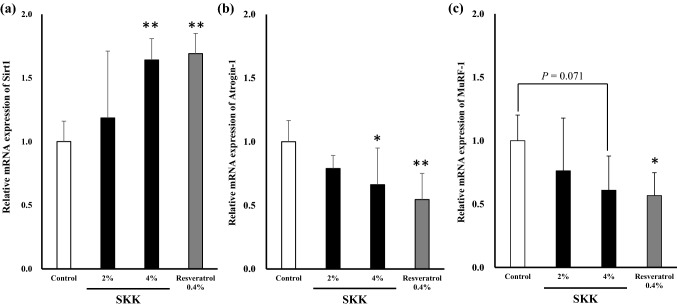
SKK increased mRNA expression of *sirt1* and decreased ubiquitin ligase mRNA expression. The gastrocnemius muscle was removed from the mice 42 days after the start of SKK administration. Next, the mRNA in the gastrocnemius was extracted. mRNA expression levels of **a** *Sirt1*, **b** Atrogin-1, and **c** MuRF-1 were measured. These data are expressed as mean ± SD (*n* = 7) and evaluated by one-way ANOVA, followed by Tukey’s test. *P < 0.05, **P < 0.01 vs. control group

We then investigated the effects of SKK on *sirt1* expression in skeletal muscle cells using qRT-PCR. Our data revealed that *sirt1* expression significantly increased in response to both 4% SKK (*P* < 0.01) and resveratrol (*P* < 0.01) when compared to the control (Fig. 6a) and these effects were shown to be significant by our one-way ANOVA (*F*_3,24_ = 9.1, *P* < 0.01).

### Ubiquitin gene expression in skeletal muscle

Total RNA was extracted from the GA samples excised from the lower limb and then used to evaluate the mRNA-based expression of the ubiquitin ligases *Atrogin-1*, *MuRF1* were measured. *Atrogin-1* expression was significantly lower in the 4% SKK (*P* = 0.023) and resveratrol groups (*P* < 0.01) when compared to the control group. While *MuRF1* showed a tendency to decrease in the 4% SKK group (*P* = 0.071) compared to the control group (Fig. 6b, c) but did not quite reach significance (Atrogin-1: *F*_3,24_ = 6.5, *P* < 0.01; MuRF1: *F*_3,24_ = 3.4, *P* = 0.035).

## Discussion

Our in vitro evaluations revealed that SKK was a strong inducer of *sirt1* transcriptional activity, and so we evaluated its effects as a potential therapeutic in an in vivo study. We found that administration of SKK extracts to KKAy mice prevented skeletal muscle atrophy and that the mRNA expression of *sirt1* was increased in these mice. In addition, SKK treatment reduced inflammatory cytokine expression, and the transcription of ubiquitin ligases *Atrogin1* and *MuRF-1*. It is thought that these factors work in combination to increase muscle mass.

Hyperglycemia leads to the overproduction of the inflammatory cytokines, increased oxidative stress, and increased insulin resistance, leading to skeletal muscle atrophy, predominantly in fast muscles [31, 32]. Here, we observed this skeletal muscle atrophy in the fast-muscle-dominant gastrocnemius and extensor digitorum longus muscles. In contrast, atrophy of these muscles was significantly reduced in mice treated with 4% SKK, while the addition of this treatment has no effect on the soleus muscle, which is mainly composed of slow muscle fibers. From these results, it was inferred that SKK could effectively reduce fast muscle-dominant muscle atrophy in KKAy mice.

Increases in the expression of the inflammatory cytokines and the resulting oxidative stress are the main causes of muscle atrophy in sarcopenic obesity [12, 13, 15]. In contrast, *Sirt1* is known to inhibit skeletal muscle atrophy by suppressing the production of inflammatory cytokines, such as TNFα and increasing the expression of antioxidant enzymes [19, 20]. Our data suggests that SKK induces the expression of *sirt1* in the gastrocnemius muscle while decreasing the expression of TNFα in the blood.

It is also widely accepted that increased inflammatory cytokines and reactive oxygen species increase the expression of ubiquitin ligase in muscle fibers and enhance muscle atrophy [21]. Here, we found that SKK significantly reduced the transcription of both *Atrogin1* and *MuRF-1* in mice treated with 4% SKK. These results suggest that SKK reduces the expression of ubiquitin ligase and alleviates skeletal muscle atrophy by increasing the expression of *sirt1* while simultaneously reducing the expression of inflammatory cytokines in these tissues. *Sirt1* has also been reported to inhibit oxidative stress [21], and the *Sirt1*-mediated reduction of oxidative stress by SKK may inhibit skeletal muscle atrophy. Furthermore, SKK decreased blood glucose levels. Decreased insulin sensitivity inhibits the growth of skeletal muscle cells and increases muscle atrophy [5, 6]. *Sirt1* is also known to improve insulin sensitivity [22, 33], but since we did not evaluate insulin sensitivity in this study, the details of this mechanism remain unknown, but it is possible that SKK suppresses muscle atrophy by improving insulin sensitivity. In addition, resveratrol, which was used as a positive control in this study, showed similar results in each of these assays, suggesting that SKK may have an effect similar to that of resveratrol. In addition, there was a trend toward improved muscle fiber cross-sectional area and wet weight in the gastrocnemius muscle, as well as reduced motor function decline in the 2% SKK diet group, but no significant improvement was observed. Therefore, we suggest that the effects of SKK are dose-dependent and cannot be achieved without sufficient concentration.

SKK is composed of seven crude herbal medicines, and the functionality of each herbal medicine has been reported. Cinnamic aldehyde, a component of *Cinnamomi Cortex*, has been reported to decrease visceral fat mass and inflammatory cytokine secretion by hypertrophic fat cells [34]. Both *Cinnamomi Cortex* and *Glycyrrhizae* R*adix*, increase the transcriptional activity of *sirt1* in C2C12 cells [24]. Baicalin, a component of *Scutellariae Radix*, has been reported to improve insulin resistance [35]. In this study, *Cinnamomi Cortex*, *Scutellariae Radix*, and *Glycyrrhizae Radix* were suggested to have high *sirt1* promoter activity in C2C12 cells. In addition, Saikosaponin, a component of *Bupleuri Radix*, has been reported to inhibit the production of TNF-α in RAW264.7 cells [36]. These suggest that the ameliorating effect of SKK on muscle atrophy is likely the result of its constituent herbal medicines, *Cinnamomi Cortex*, *Glycyrrhizae Radix*, *Scutellariae Radix*, and *Bupleuri Radix*. In this study, we focused on TNF-α as an inflammatory cytokine for evaluation. However, there are various other inflammatory cytokines, such as IL-6, which are related to insulin resistance apart from TNF-α [37]. The effects of SKK on other inflammatory cytokines need to be investigated in future studies.”

## Conclusion

The results of this study suggest that SKK inhibits muscle atrophy in KKAy mice. Skeletal muscle atrophy in KKAy mice closely resembles the symptoms of sarcopenic obesity, and SKK is expected to inhibit the progression of sarcopenia symptoms in clinical practice.

## Supplementary Information

Below is the link to the electronic supplementary material.

Supplementary file1 (PPTX 49 kb)

Supplementary file2 (PPTX 693 kb)

Supplementary file3 (PPTX 41 kb)

## References

[1] Bassett DR, Pucher J, Buehler R, Thompson DL, Crouter SE (2008). Walking, cycling, and obesity rates in Europe, North America, and Australia. J Phys Act Health.

[2] Drewnowski A (2004). Obesity and the food environment: dietary energy density and diet costs. Am J Prev Med.

[3] Choi KM (2016). Sarcopenia and sarcopenic obesity. Korean J Intern Med.

[4] Baumgartner RN, Wayne SJ, Waters DL, Janssen I, Gallagher D, Morley JE (2004). Sarcopenic obesity predicts instrumental activities of daily living disability in the elderly. Obes Res.

[5] Kim TN, Park MS, Lim KI, Choi HY, Yang SJ, Yoo HJ, Kang HJ, Song W, Choi H, Baik SH, Choi DS, Choi KM (2013). Relationships between sarcopenic obesity and insulin resistance, inflammation, and vitamin D status: the Korean Sarcopenic Obesity Study. Clin Endocrinol (Oxf).

[6] Srikanthan P, Hevener AL, Karlamangla AS (2010). Sarcopenia exacerbates obesity-associated insulin resistance and dysglycemia: findings from the national health and nutrition examination survey III. PLoS ONE.

[7] Stenholm S, Harris TB, Rantanen T, Visser M, Kritchevsky SB, Ferrucci L (2008). Sarcopenic obesity: definition, cause and consequences. Curr Opin Clin Nutr Metab Care.

[8] Schrager MA, Metter EJ, Simonsick E, Ble A, Bandinelli S, Lauretani F, Ferrucci L (2007). Sarcopenic obesity and inflammation in the InCHIANTI study. J Appl Physiol.

[9] Vincent HK, Raiser SN, Vincent KR (2012). The aging musculoskeletal system and obesity-related considerations with exercise. Ageing Res Rev.

[10] Park HS, Park JY, Yu R (2005). Relationship of obesity and visceral adiposity with serum concentrations of CRP, TNF-α and IL-6. Diabetes Res Clin Pract.

[11] Kern PA, Ranganathan S, Li C, Wood L, Ranganathan G (2001). Adipose tissue tumor necrosis factor and interleukin-6 expression in human obesity and insulin resistance. Am J Physiol Endocrinol Metab.

[12] Helenius M, Hänninen M, Lehtinen SK, Salminen A (1996). Aging-induced up-regulation of nuclear binding activities of oxidative stress responsive NF-kB transcription factor in mouse cardiac muscle. J Mol Cell Cardiol.

[13] Li H, Malhotra S, Kumar A (2008). Nuclear factor-kappa B signaling in skeletal muscle atrophy. J Mol Med (Berl).

[14] Furukawa S, Fujita T, Shimabukuro M, Iwaki M, Yamada Y, Nakajima Y, Nakayama O, Makishima M, Matsuda M, Shimomura I (2004). Increased oxidative stress in obesity and its impact on metabolic syndrome. J Clin Invest.

[15] Park SW, Goodpaster BH, Strotmeyer ES, de Rekeneire N, Harris TB, Schwartz AV, Tylavsky FA, Newman AB (2006). Decreased muscle strength and quality in older adults with type 2 diabetes: the health, aging, and body composition study. Diabetes.

[16] Powers SK, Kavazis AN, McClung JM (2007). Oxidative stress and disuse muscle atrophy. J Appl Physiol.

[17] Suto J, Matsuura S, Imamura K, Yamanaka H, Sekikawa K (1998). Genetic analysis of non-insulin-dependent diabetes mellitus in KK and KK-Ay mice. Eur J Endocrinol.

[18] Takamura Y, Nomura M, Uchiyama A, Fujita S (2017). Effects of aerobic exercise combined with panaxatriol derived from ginseng on insulin resistance and skeletal muscle mass in type 2 diabetic mice. J Nutr Sci Vitaminol (Tokyo).

[19] Tonkin J, Villarroya F, Puri PL, Vinciguerra M (2012). SIRT1 signaling as potential modulator of skeletal muscle diseases. Curr Opin Pharmacol.

[20] Liao ZY, Chen JL, Xiao MH, Sun Y, Zhao YX, Pu D, Lv AK, Wang ML, Zhou J, Zhu SY, Zhao KX, Xiao Q (2017). The effect of exercise, resveratrol or their combination on sarcopenia in aged rats *via* regulation of AMPK/Sirt1 pathway. Exp Gerontol.

[21] Lee D, Goldberg AL (2013). SIRT1 protein, by blocking the activities of transcription factors FoxO1 and FoxO3, inhibits muscle atrophy and promotes muscle growth. J Biol Chem.

[22] Cao Y, Jiang X, Ma H, Wang Y, Xue P, Liu Y (2016). SIRT1 and insulin resistance. J Diabetes Complicat.

[23] Kishida Y, Kagawa S, Arimitsu J, Nakanishi M, Sakashita N, Otsuka S, Yoshikawa H, Hagihara K (2015). Go-sha-jinki-Gan (GJG), a traditional Japanese herbal medicine, protects against sarcopenia in senescence-accelerated mice. Phytomedicine.

[24] Ishida T, Iizuka M, Ou Y, Morisawa S, Hirata A, Yagi Y, Jobu K, Morita Y, Miyamura M (2019). Juzentaihoto hot water extract alleviates muscle atrophy and improves motor function in streptozotocin-induced diabetic oxidative stress mice. J Nat Med.

[25] Morita Y, Ishida T, Morisawa S, Jobu K, Ou Y, Fujita H, Hanazaki K, Miyamura M (2021). Juzentaihoto suppresses muscle atrophy and decreased motor function in SAMP8 mice. Biol Pharm Bull.

[26] Uto NS, Amitani H, Atobe Y, Sameshima Y, Sakaki M, Rokot N, Ataka K, Amitani M, Inui A (2018). Herbal medicine Ninjin’yoeito in the treatment of sarcopenia and frailty. Front Nutr.

[27] The Japanese pharmacopoeia (2016) (JP17), 17th ed. http://jpdb.nihs.go.jp/jp17e/. Ministry of Health, Labour and Welfare, Japan

[28] Nemoto S, Fergusson MM, Finkel T (2004). Nutrient availability regulates SIRT1 through a forkhead-dependent pathway. Science.

[29] Zhu W, Chen S, Li Z, Zhao X, Li W, Sun Y, Zhang Z, Ling W, Feng X (2014). Effects and mechanisms of resveratrol on the amelioration of oxidative stress and hepatic steatosis in KKAy mice. Nutr Metab (Lond).

[30] Chen S, Li J, Zhang Z, Li W, Sun Y, Zhang Q, Feng X, Zhu W (2012). Effects of resveratrol on the amelioration of insulin resistance in KKAy mice. Can J Physiol Pharmacol.

[31] Ciciliot S, Rossi AC, Dyar KA, Blaauw B, Schiaffino S (2013). Muscle type and fiber type specificity in muscle wasting. Int J Biochem Cell Biol.

[32] Price SR, Bailey JL, Wang X, Jurkovitz C, England BK, Ding X, Phillips LS, Mitch WE (1996). Muscle wasting in insulinopenic rats results from activation of the ATP-dependent, ubiquitin-proteasome proteolytic pathway by a mechanism including gene transcription. J Clin Invest.

[33] Kitada M, Ogura Y, Monno I, Koya D (2019). Sirtuins and Type 2 diabetes: Role in inflammation, oxidative stress, and mitochondrial function. Front Endocrinol (Lausanne).

[34] Neto JGO, Boechat SK, Romão JS, Pazos-Moura CC, Oliveira KJ (2020). Treatment with cinnamaldehyde reduces the visceral adiposity and regulates lipid metabolism, autophagy and endoplasmic reticulum stress in the liver of a rat model of early obesity. J Nutr Biochem.

[35] Fang P, Sun Y, Gu X, Shi M, Bo P, Zhang Z, Bu L (2019). Baicalin ameliorates hepatic insulin resistance and gluconeogenic activity through inhibition of p38 MAPK/PGC-1α pathway. Phytomedicine.

[36] Lu CN, Yuan ZG, Zhang XL, Yan R, Zhao YQ, Liao M, Chen JX (2012). Saikosaponin a and its epimer saikosaponin d exhibit anti-inflammatory activity by suppressing activation of NF-κB signaling pathway. Int Immunopharmacol.

[37] Rehman K, Akash MSH, Liaqat A, Kamal S, Qadir MI, Rasul A (2017). Role of interleukin-6 in development of insulin resistance and type 2 diabetes mellitus. Crit Rev Eukaryot Gene Expr.

