# Supplementary material for: Saikokeishikankyoto extract alleviates muscle atrophy in KKAy mice
Source: J Nat Med. 2022 Jan 8;76(2):379–88. doi: 10.1007/s11418-021-01590-2 (PMC8858927; doi:10.1007/s11418-021-01590-2)
Supplement: Supplementary file 1 — Supplementary file1 (PPTX 49 kb) [file 11418_2021_1590_MOESM1_ESM.pptx]

## Slide 1
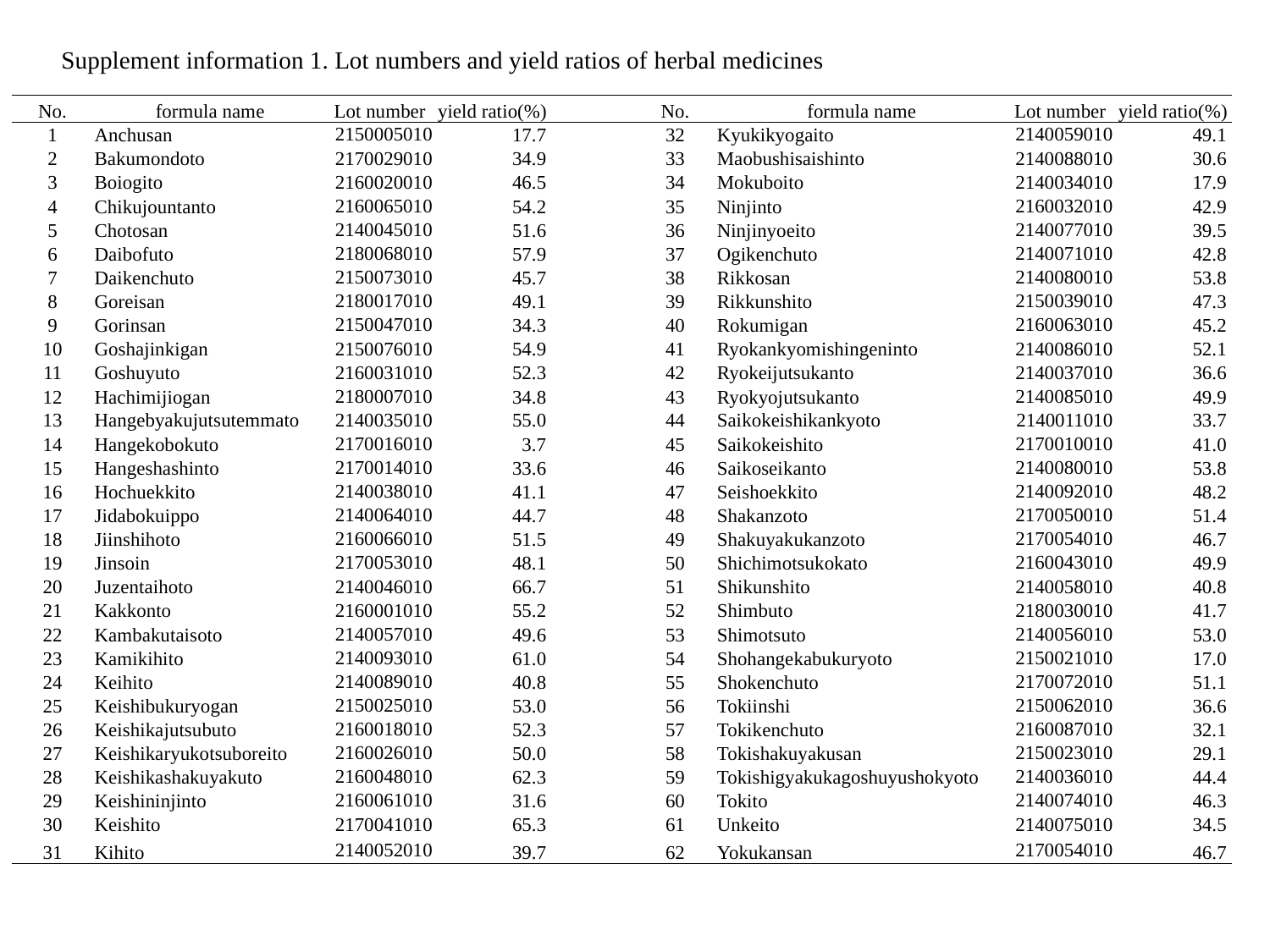

Supplement information 1. Lot numbers and yield ratios of herbal medicines
| No. | formula name | Lot number | yield ratio(%) | | No. | formula name | Lot number | yield ratio(%) |
| --- | --- | --- | --- | --- | --- | --- | --- | --- |
| 1 | Anchusan | 2150005010 | 17.7 | | 32 | Kyukikyogaito | 2140059010 | 49.1 |
| 2 | Bakumondoto | 2170029010 | 34.9 | | 33 | Maobushisaishinto | 2140088010 | 30.6 |
| 3 | Boiogito | 2160020010 | 46.5 | | 34 | Mokuboito | 2140034010 | 17.9 |
| 4 | Chikujountanto | 2160065010 | 54.2 | | 35 | Ninjinto | 2160032010 | 42.9 |
| 5 | Chotosan | 2140045010 | 51.6 | | 36 | Ninjinyoeito | 2140077010 | 39.5 |
| 6 | Daibofuto | 2180068010 | 57.9 | | 37 | Ogikenchuto | 2140071010 | 42.8 |
| 7 | Daikenchuto | 2150073010 | 45.7 | | 38 | Rikkosan | 2140080010 | 53.8 |
| 8 | Goreisan | 2180017010 | 49.1 | | 39 | Rikkunshito | 2150039010 | 47.3 |
| 9 | Gorinsan | 2150047010 | 34.3 | | 40 | Rokumigan | 2160063010 | 45.2 |
| 10 | Goshajinkigan | 2150076010 | 54.9 | | 41 | Ryokankyomishingeninto | 2140086010 | 52.1 |
| 11 | Goshuyuto | 2160031010 | 52.3 | | 42 | Ryokeijutsukanto | 2140037010 | 36.6 |
| 12 | Hachimijiogan | 2180007010 | 34.8 | | 43 | Ryokyojutsukanto | 2140085010 | 49.9 |
| 13 | Hangebyakujutsutemmato | 2140035010 | 55.0 | | 44 | Saikokeishikankyoto | 2140011010 | 33.7 |
| 14 | Hangekobokuto | 2170016010 | 3.7 | | 45 | Saikokeishito | 2170010010 | 41.0 |
| 15 | Hangeshashinto | 2170014010 | 33.6 | | 46 | Saikoseikanto | 2140080010 | 53.8 |
| 16 | Hochuekkito | 2140038010 | 41.1 | | 47 | Seishoekkito | 2140092010 | 48.2 |
| 17 | Jidabokuippo | 2140064010 | 44.7 | | 48 | Shakanzoto | 2170050010 | 51.4 |
| 18 | Jiinshihoto | 2160066010 | 51.5 | | 49 | Shakuyakukanzoto | 2170054010 | 46.7 |
| 19 | Jinsoin | 2170053010 | 48.1 | | 50 | Shichimotsukokato | 2160043010 | 49.9 |
| 20 | Juzentaihoto | 2140046010 | 66.7 | | 51 | Shikunshito | 2140058010 | 40.8 |
| 21 | Kakkonto | 2160001010 | 55.2 | | 52 | Shimbuto | 2180030010 | 41.7 |
| 22 | Kambakutaisoto | 2140057010 | 49.6 | | 53 | Shimotsuto | 2140056010 | 53.0 |
| 23 | Kamikihito | 2140093010 | 61.0 | | 54 | Shohangekabukuryoto | 2150021010 | 17.0 |
| 24 | Keihito | 2140089010 | 40.8 | | 55 | Shokenchuto | 2170072010 | 51.1 |
| 25 | Keishibukuryogan | 2150025010 | 53.0 | | 56 | Tokiinshi | 2150062010 | 36.6 |
| 26 | Keishikajutsubuto | 2160018010 | 52.3 | | 57 | Tokikenchuto | 2160087010 | 32.1 |
| 27 | Keishikaryukotsuboreito | 2160026010 | 50.0 | | 58 | Tokishakuyakusan | 2150023010 | 29.1 |
| 28 | Keishikashakuyakuto | 2160048010 | 62.3 | | 59 | Tokishigyakukagoshuyushokyoto | 2140036010 | 44.4 |
| 29 | Keishininjinto | 2160061010 | 31.6 | | 60 | Tokito | 2140074010 | 46.3 |
| 30 | Keishito | 2170041010 | 65.3 | | 61 | Unkeito | 2140075010 | 34.5 |
| 31 | Kihito | 2140052010 | 39.7 | | 62 | Yokukansan | 2170054010 | 46.7 |
