# Supplementary material for: Saikokeishikankyoto extract alleviates muscle atrophy in KKAy mice
Source: J Nat Med. 2022 Jan 8;76(2):379–88. doi: 10.1007/s11418-021-01590-2 (PMC8858927; doi:10.1007/s11418-021-01590-2)
Supplement: Supplementary file 2 — Supplementary file2 (PPTX 693 kb) [file 11418_2021_1590_MOESM2_ESM.pptx]

## Slide 1
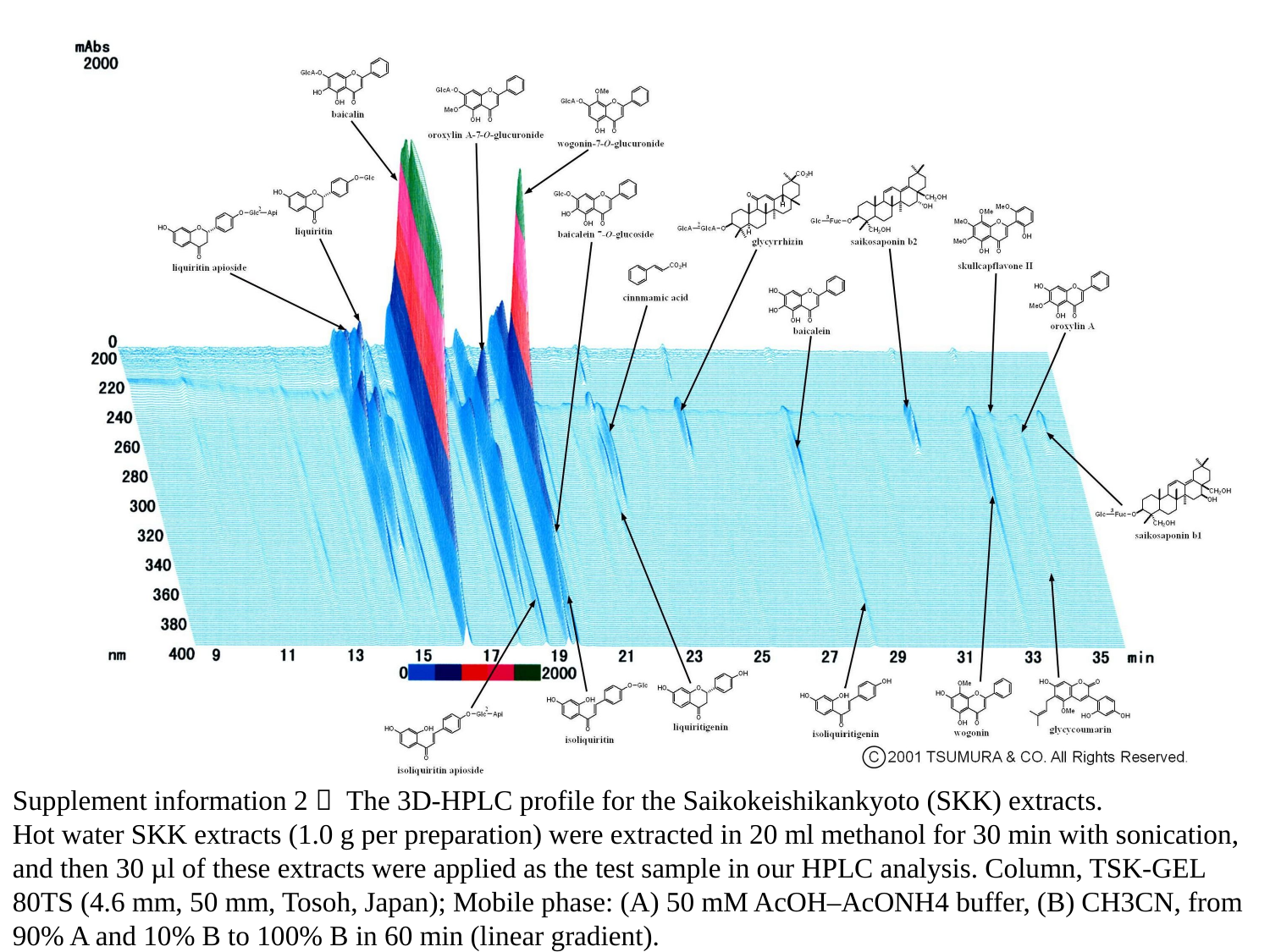

Supplement information 2： The 3D-HPLC profile for the Saikokeishikankyoto (SKK) extracts.
Hot water SKK extracts (1.0 g per preparation) were extracted in 20 ml methanol for 30 min with sonication, and then 30 µl of these extracts were applied as the test sample in our HPLC analysis. Column, TSK-GEL 80TS (4.6 mm, 50 mm, Tosoh, Japan); Mobile phase: (A) 50 mM AcOH–AcONH4 buffer, (B) CH3CN, from 90% A and 10% B to 100% B in 60 min (linear gradient).
