# Supplementary material for: Saikokeishikankyoto extract alleviates muscle atrophy in KKAy mice
Source: J Nat Med. 2022 Jan 8;76(2):379–88. doi: 10.1007/s11418-021-01590-2 (PMC8858927; doi:10.1007/s11418-021-01590-2)
Supplement: Supplementary file 3 — Supplementary file3 (PPTX 41 kb) [file 11418_2021_1590_MOESM3_ESM.pptx]

## Slide 1
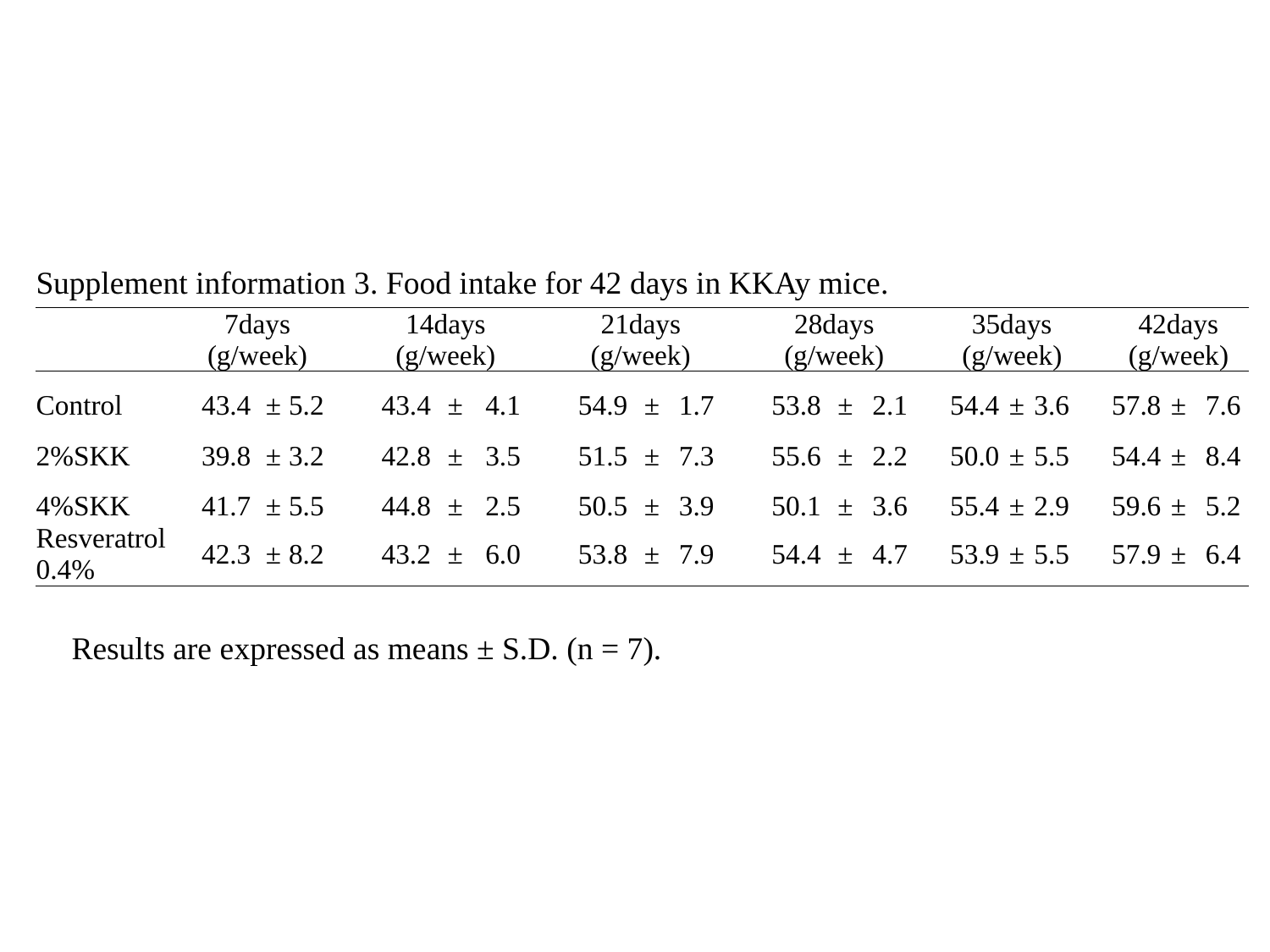

Supplement information 3. Food intake for 42 days in KKAy mice.
| | 7days (g/week) | | | | 14days (g/week) | | | | 21days (g/week) | | | | 28days (g/week) | | | | 35days (g/week) | | | | 42days (g/week) | | |
| --- | --- | --- | --- | --- | --- | --- | --- | --- | --- | --- | --- | --- | --- | --- | --- | --- | --- | --- | --- | --- | --- | --- | --- |
| Control | 43.4 | ± | 5.2 | | 43.4 | ± | 4.1 | | 54.9 | ± | 1.7 | | 53.8 | ± | 2.1 | | 54.4 | ± | 3.6 | | 57.8 | ± | 7.6 |
| 2%SKK | 39.8 | ± | 3.2 | | 42.8 | ± | 3.5 | | 51.5 | ± | 7.3 | | 55.6 | ± | 2.2 | | 50.0 | ± | 5.5 | | 54.4 | ± | 8.4 |
| 4%SKK | 41.7 | ± | 5.5 | | 44.8 | ± | 2.5 | | 50.5 | ± | 3.9 | | 50.1 | ± | 3.6 | | 55.4 | ± | 2.9 | | 59.6 | ± | 5.2 |
| Resveratrol 0.4% | 42.3 | ± | 8.2 | | 43.2 | ± | 6.0 | | 53.8 | ± | 7.9 | | 54.4 | ± | 4.7 | | 53.9 | ± | 5.5 | | 57.9 | ± | 6.4 |
Results are expressed as means ± S.D. (n = 7).
